# The first Silurian horseshoe crab reveals details of the xiphosuran ground plan

**DOI:** 10.1098/rspb.2025.0874

**Published:** 2025-06-18

**Authors:** James C. Lamsdell

**Affiliations:** ^1^ Department of Geology and Geography, West Virginia University, Morgantown, WV 26506, USA

**Keywords:** *Ciurcalimulus*, ground plan, horseshoe crab, laser stimulated fluorescence, Silurian, Xiphosura, Xiphosurida

## Abstract

Horseshoe crabs are an ancient lineage with an evolutionary history stretching back 450 million years and are generally considered to be examples of ‘living fossils’ exhibiting slow rates of evolution. Despite this reputation, relatively little is known of the early evolution of the group, with only two species described from the Ordovician and a subsequent 80-million-year gap in their fossil record until xiphosurids appear in the Late Devonian. Furthermore, all described Ordovician species are assigned to a single genus, with their close phylogenetic relatedness rendering it unclear whether their morphology is representative of the horseshoe crab ground pattern or an independently derived condition. Here, a new species of horseshoe crab is described from the Silurian of Indiana, USA. The new species bridges the temporal gap in the xiphosuran fossil record and has an overall morphology similar to that of the Ordovician taxa. These new data provide critical information on the ancestral morphology of horseshoe crabs, showing that xiphosurids evolved from forms with a fused thoracetron exhibiting axial segment boundaries, and demonstrate the persistence of basal Xiphosura into the Silurian. Laser-stimulated fluorescence is also shown to be an effective method for studying and imaging arthropod fossils exhibiting challenging preservation.

## 1. Introduction

Horseshoe crabs are aquatic chelicerate arthropods defined by the fusion of their body segments into a thoracetron [1]. Four extant species are known and exhibit a disjunct geographical distribution, with one species occurring in the western Atlantic (ranging from the east coast of Canada to the Gulf of Mexico) and three in the western Pacific and northeast Indian Oceans (extending from the south of Japan to the east coast of India) [2]. The group is famous as an example of an evolutionary conservative lineage and is considered to comprise archetypal ‘living fossils’ [3–5], although more recent work has demonstrated repeated ecological transitions within the group to be associated with the development of extreme morphologies [6–8]. Horseshoe crabs have a long evolutionary history stretching back to the Late Ordovician (approx. 450 Ma) with two species described from North America [9,10] and another, slightly older (Lower Ordovician, approx. 480 Ma) species reported but awaiting formal description from Morocco [11]. The origins and early evolution of horseshoe crabs are poorly known, however, with an 80 Myr gap between these Ordovician species and the first record of Xiphosurida (horseshoe crabs that have reduced their postabdomen to a single segment) in the Late Devonian (Famennian, approx. 370 Ma) [12,13]. This lack of a fossil record for horseshoe crabs in the Silurian, a time during which other aquatic chelicerate groups were rapidly diversifying [14], makes it difficult to determine the timing of the origin of xiphosurids and to what extent the end Ordovician mass extinction and Silurian ecosystem recovery influenced horseshoe crab evolution. Furthermore, the close phylogenetic relatedness of the described Ordovician species (which both resolve within the genus *Lunataspis*) makes it unclear whether their morphology is typical of the horseshoe crab ground pattern or representative of an independently derived condition of a single genus.

Here, I report on the first true horseshoe crab (total group Xiphosura) from the Silurian based on a single specimen from the Kokomo Member of Indiana, USA. The specimen is determined to be a new genus and species, named *Ciurcalimulus discobolus* gen. et sp. nov., which phylogenetic analysis resolves as the sister taxon to Xiphosurida. As such, comparison between the new species and *Lunataspis* allows determination of the horseshoe crab ground pattern for the first time. This discovery demonstrates that non-xiphosurid horseshoe crabs persisted beyond the end Ordovician mass extinction event and that Laurentia was likely an important region in early horseshoe crab evolution.

## 2. Material and methods

The only known specimen of *Ciurcalimulus discobolus* is deposited in the Yale Peabody Museum (YPM) Division of Invertebrate Paleontology under the specimen number YPM IP 548961. Taxonomic acts for this publication are registered at ZooBank under Life Science Identifier urn:lsid:zoobank.org:pub:1D3E204C−3E4A−4D35−8095−1695E4A6C358.

### (a) Geological setting

The new specimen (YPM IP 548961) was found in 1975 by Samuel J. Ciurca, Jr in the Kokomo Member of the Wabash Formation from Indiana, USA. The Kokomo Member comprises up to 30 m of finely laminated dark dolostones [15] and is considered to be Silurian (Upper Ludlow, approx. 424 Ma) in age based on conodont data [16], correlated with Unit D of the Salina Group in Michigan [17]. The Kokomo localities are primarily known for their endemic eurypterid fauna [18–22], which occur in a single horizon and are recognized to represent a mass mortality event [23], although a variety of algae co-occur with the eurypterids [24] and brachiopods are found alongside corals near the top of the member in what is sometimes referred to as the brachiopod horizon [25]. The xiphosuran is derived from the eurypterid horizon and is preserved similarly to the eurypterids, which are compression fossils with carbonized cuticle [26].

The environment of deposition of the Kokomo Member is spatially and temporally variable, with desiccation cracks in parts of the member suggesting a supratidal environment [27], while the presence of corals near the top of the member indicates marine conditions. Neither occurs in the eurypterid horizon, which is interpreted as a restricted, low-energy environment within a nearshore marine setting.

### (b) Imaging

The specimen was photographed both dry and immersed in ethanol under polarized light, ultraviolet light and laser-stimulated fluorescence [28]. Laser-stimulated fluorescence images were produced using a 500 mW, 447 nm blue laser (LRD−0447 Collimated Diode Laser System) with a MidOpt LP500−67 yellow longpass filter attached to the camera lens and a 500 mW, 532 nm green laser (MGL-FN−532 Diode-Pumped Solid-State Laser System) with a MidOpt LP550−67 orange longpass filter attached to the camera lens. Ultraviolet images were produced using a Nightsea Dual Fluorescent Protein flashlight. All images were taken with a Canon EOS 5D camera mounting a Canon EF 100 mm *f*/2.8 IS USM macro lens or a Leica EZ4 HD Digital Stereo Microscope.

### (c) Phylogenetic analysis

To assess the phylogenetic position of the new species, a phylogenetic analysis with tree inference performed using maximum parsimony was conducted based on a modified matrix derived from previous analyses of chelicerate relationships [1,2,6,7,29–32]. The resulting matrix comprises 257 characters coded for 161 taxa and includes a comprehensive sampling of xiphosurans alongside representatives of other euchelicerate groups (eurypterids, chasmataspidids, arachnids), stem euchelicerates (‘synziphosurines’ [29], offacolids [33,34], habeliids [35], mollisoniids [36]) and pycnogonids. The analysis is rooted on the megacheiran arthropod *Yohoia*, megacheirans being generally accepted as either the sister group to Chelicerata [37,38] or Chelicerata and Mandibulata [39,40]. The phylogenetic matrix is available in the online MorphoBank database [41] under the project code p1253. The analysis was performed using TNT [42], employing 100 000 random addition sequences with all characters unordered and of equal weight [43], each followed by tree bisection–reconnection branch swapping. Bootstrap [44] branch support values were calculated with 50% resampling for 1000 repetitions.

## 3. Results

### (a) Systematic palaeontology

Arthropoda Gravenhorst, 1843 [45]

Chelicerata Heymons, 1901 [46]

Xiphosura Latreille, 1802 [47]

*Ciurcalimulus discobolus* gen. et sp. nov.

LSID: urn:lsid:zoobank:act: A49E2CE6−A3E8−4162 A933−7BC2DA393862 and urn:lsid:zoobank.org:act:5485C4F4−5D26−4F20−9806−4AB982781591.

*Etymology*. The genus is named for the late Sam Ciurca, a prolific collector and avocational palaeontologist who discovered the specimen in 1975. The species name refers to the ancient Greek sculpture by Myron in reference to the extremely rounded, discus-like form of the prosoma and thoracetron.

**Figure 1.**
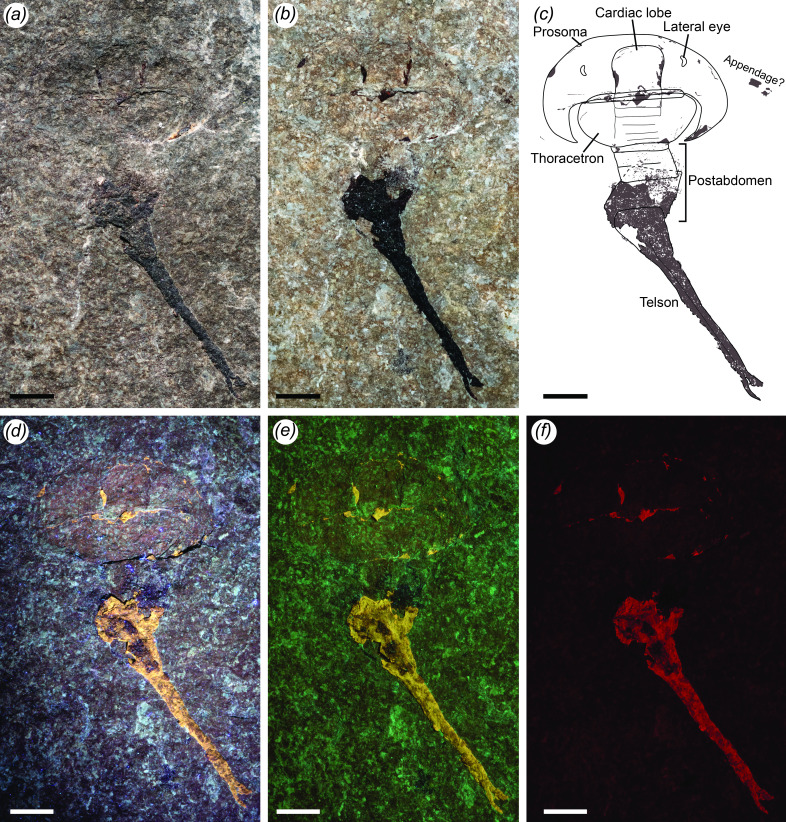
*Ciurcalimulus discobolus* gen. et sp. nov., holotype (YPM IP 548961). (*a*) Imaged dry under polarized light, showing specimen relief. (*b*) Imaged immersed in ethanol under polarized light, providing greater contrast of the carbonized cuticle. (*c*) Interpretive drawing of specimen. Carbonized cuticle is shown in brown, damaged or incomplete margins by dashed lines. (*d*) Imaged under ultraviolet light, exhibiting fluorescence of both the carbonized cuticle and to a lesser extent the exfoliated regions of the fossil, affording the clearest view of the segment boundaries in the thoracetron axis. (*e*) Imaged under 447 nm blue laser, with carbonized cuticle and exfoliated regions again both fluorescing. The outline of the fossil is most readily apparent here as is the morphology of the cardiac lobe. (*f*) Imaged under 532 nm green laser, clearly showing the location of carbonized cuticle via strong fluorescence. Scale bars = 5 mm.

**Figure 2.**
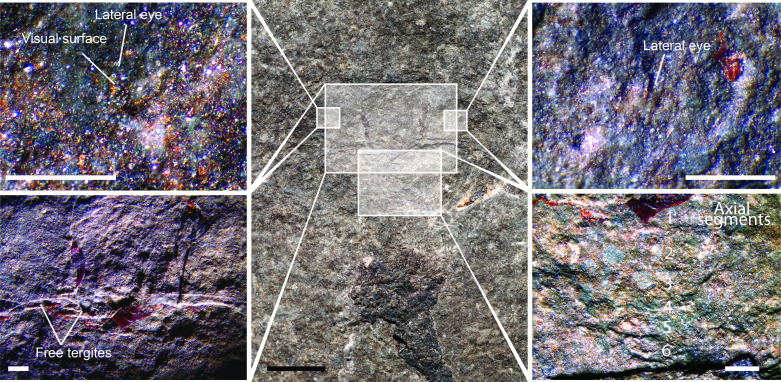
*Ciurcalimulus discobolus* gen. et sp. nov., holotype (YPM IP 548961). Details of the specimen photographed under normal light, with the magnified regions indicated. Scale bars of magnifications = 1 mm, 5 mm on overview image.

*Holotype*. YPM IP 548961 (Yale Peabody Museum, Invertebrate Paleontology), complete specimen in dorsal view preserving the prosomal carapace, thoracetron, postabdomen and telson (figures 1 and 2).

*Location and age*. Kokomo Member of the Wabash Formation, Indiana, USA (40°28'22.1"N 86°10'47.8"W); Silurian (Upper Ludlow, approx. 424 Ma).

*Diagnosis*. Xiphosuran with semicircular prosomal carapace bearing curved genal spines that reach almost to thoracetron posterior; prosomal carapace bearing crescentic lateral eyes located centrimesially; prosomal cardiac lobe quadrate with rounded anterior, well expressed with margins defined by furrows; anterior two opisthosomal segments short and freely articulating; thoracetron semicircular, approximately equal in length to the prosomal carapace, comprising up to six fused body segments with segmental boundaries expressed axially; postabdomen comprising up to five free articulating segments including an elongated pretelson; telson equal in length to entire body, lanceolate in shape with bifurcate termination.

### (b) Description

*Ciurcalimulus discobolus* is known from a single specimen (YPM IP 548961), which preserves the dorsal prosomal carapace, opisthosoma, and telson in its entirety (figure 1). As with other arthropods known from the Kokomo Member [22], the specimen is dorsoventrally compressed and preserves little in the way of original relief. Some remnants of carbonized organic cuticle are present, primarily on the pretelson and telson but also within grooves delineating the margins of structures within the prosoma and thoracetron, which fluoresce under ultraviolet and laser stimulation (figure 1*d*–*f*). Many of the available details, such as the segmental boundaries within the thoracetron axis and well-defined cardiac lobe margin, are only visible under laser stimulation; this once again demonstrates the utility of laser-stimulated fluorescence in revealing details of unmineralized anatomical structures [48], in this case in an arthropod fossil exhibiting challenging preservation. The specimen has a total length of 44.8 mm but exhibits marked curvature of the postabdomen as well as telescoping of the pretelson and telson, all of which are indicative of the specimen being a moult [49,50].

The prosomal carapace is semicircular in shape, 6.2 mm long (not including the genal spines) and 20.5 mm wide at its base. The genal spines contribute an additional 5.8 mm in length, are 2.7 mm wide at their base, and narrow evenly posteriorly following the curvature of the thoracetron, with a groove running along their interior margin. A well defined cardiac lobe is located centrally at the carapace posterior, broadly quadrate in shape, with a length of 5.0 mm and a posterior width of 4.9 mm, expanding to 5.2 mm at its rounded anterior. The locations of a pair of lateral eyes are faintly indicated by discoloured depressions on the carapace (figure 2); the crescentic visual surfaces are located centrimesially on the carapace, 2.7 mm from the posterior and 1.7 mm from the lateral margin and are 0.5 mm in width by 1.5 mm in length. Neither the median eyes nor any ophthalmic ridges associated with the lateral eyes are preserved, although this is likely due to the fine scale of these structures and the limits of preservation on the fossil. To the right of the prosomal carapace is faintly preserved carbonized organic cuticle that may represent the distal podomeres of a prosomal appendage. The shape of the cuticle corresponds to what would be expected of a chelate appendage; however, it is not possible to make any definitive statements regarding the structure.

Posterior to the prosomal carapace are two tergites with clearly defined segmental boundaries (figure 2), indicating that they are freely articulating [1,51]. The first tergite has a length of 0.5 mm and a width of 10.2 mm, with a clearly defined 5.0 mm wide axial region. The second tergite has a length of 0.6 mm with a width of 10.5 mm and a 5.2 mm wide axis. Subsequent to these tergites is a semicircular thoracetron with a length of 5.1 mm and an anterior width of 14.1 mm, narrowing to 7.2 mm at its posterior. An axial region is faintly visible running down the centre of the thoracetron with the boundaries of individual segments present within it, most clearly seen under ultraviolet light and 447 nm blue laser (figure 1*d*,*e*). The total number of segments within the thoracetron is difficult to ascertain with certainty but appears to be six (figure 2); three segment axes each with a length of 1.0 mm can be seen followed by up to three smaller segment axes occupying the posterior 2 mm of the thoracetron. These posterior segments appear to have an average length of around 0.5 mm. The axial region itself widens from 5.2 mm in width anteriorly to 6.3 mm in width posteriorly. The thoracetron lacks visible segmental boundaries outside of the axial region, nor is there any indication of a demarcated margin.

A postabdomen composed of up to four or five freely articulating segments is located posterior to the thoracetron. The first few segments are kinked toward the right and are 6.3 mm in width; the total number of segments is again difficult to ascertain with certainty, but there appear to be three with an average length of 1.2 mm. Posterior to these is potentially a fourth segment followed by the pretelson, which partially telescoped into the preceding segments on the left side, resulting in the pretelson being angled in the opposite direction to the rest of the postabdomen. The pretelson is elongated, being 4.7 mm long, and moderately expanded to a width of 7.0 mm. Small epimera may be present at the posterolateral margins. Subsequent to the pretelson is an elongate, 28.5 mm long, lanceolate telson with a proximal width of 5.3 mm. The telson is partially telescoped into the pretelson and is angled back towards to the right. A small dorsal ridge runs the length of the telson. At the termination of the ridge the telson bifurcates into two short prongs, the right one of which has been damaged. The prong on the left is preserved in its entirety.

### (c) Phylogenetic placement

**Figure 3.**
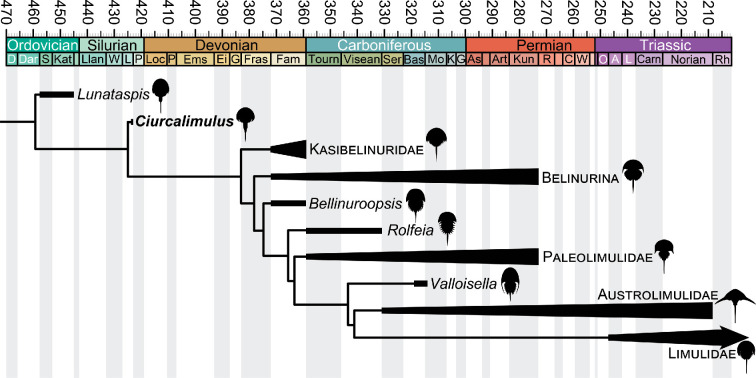
Simplified phylogeny of Xiphosura showing the phylogenetic position of *Ciurcalimulus discobolus*. For full details of the phylogeny, including branch supports, see electronic supplementary material, figure S1. Stages are shown within each Period, with names and ages as per the International Commission on Stratigraphy.

Phylogenetic analysis resulted in two most parsimonious trees of 727 steps, the strict consensus of which (figure 3) is in accordance with the results of previous analyses [1,2,6,7,29–31]. *Ciurcalimulus discobolus* resolves within Xiphosura, intermediate between the Ordovician *Lunataspis* and the Devonian–Recent Xiphosurida, in congruence with its Silurian age. An assignment of *Ciurcalimulus* to Xiphosura is supported primarily by its possession of a fused thoracetron and reinforced by the presence of a groove along the interior margin of the carapace genal spine and elongated telson in excess of half the total body length. The new species is excluded from Xiphosurida owing to its retention of a freely articulating postabdomen composed of more than a single segment, a position further supported by the lack of a well developed ophthalmic ridge. A sister-group relationship of *Ciurcalimulus* to Xiphosurida is supported by the possession of a well defined cardiac lobe that extends beyond the posterior half of the prosomal carapace.

## 4. Discussion

### (a) Comparison with other early Palaeozoic Xiphosura

*Ciurcalimulus discobolus* is readily distinguished from other early Palaeozoic horseshoe crabs through its unique combination of characteristics unknown among other species. The bifurcating telson termination of *Ciurcalimulus* is currently unique among Xiphosura and serves as an autapomorphy for the taxon. The species is clearly distinct from the North American Devonian xiphosurids *Patesia* and *Pickettia* owing to the lack of a well developed ophthalmic ridge, which is present in both Devonian genera, and in the retention of a multisegmented postabdomen in *Ciurcalimulus*, a characteristic that also separates the Kokomo xiphosuran from all xiphosurids. *Ciurcalimulus* also exhibits no indication of expressed segmental boundaries in the lateral regions of the thoracetron, whereas both Devonian genera exhibit obvious segment boundaries across the entirety of their thoracetron [1,12,13].

*Ciurcalimulus* most closely resembles the Ordovician *Lunataspis* species in possessing a heavily rounded prosomal carapace and a semicircular thoracetron lacking lateral segment boundary expression or tergopleural projections [9,10] as well as a multisegmented postabdomen. However, the new genus is distinguished from *Lunataspis* in lacking axial nodes on the thoracetron and the absence of a thoracetron marginal rim defined dorsally by a furrow, both of which are present in the Ordovician species. Neither *Lunataspis* species exhibits a well defined cardiac lobe on the prosomal carapace, instead possessing a short cardiac lobe restricted to the posterior region of the carapace and evidenced by only a faint topological swelling of the carapace surface. This is contrasted strongly by the cardiac lobe in *Ciurcalimulus*, which extends well onto the anterior half of the prosomal carapace and is strongly defined by a set of furrows as in xiphosurids. The form of the cardiac lobe also differentiates *Ciurcalimulus* from the undescribed xiphosuran from Fezouata, which exhibits no obvious cardiac lobe demarcation [1]. The Fezouata species is further distinct from *Ciurcalimulus* in possessing a more angular thoracetron and undeveloped genal regions of the prosomal carapace [1,11].

### (b) The horseshoe crab ground pattern

The resolution of *Ciurcalimulus* as phylogenetically intermediate between *Lunataspis* and Xiphosurida affords an opportunity to explore the likely ground pattern of horseshoe crabs for the first time, with traits shared between *Lunataspis*, the undescribed Fezouata species and *Ciurcalimulus* presumably indicating a common ancestral state. Many traits considered characteristic of horseshoe crabs are present in their ground pattern: a semicircular prosomal carapace with long genal spines, a thoracetron comprising at least six fused opisthosomal segments, and an elongated telson at least half the total body length. Several characteristics previously only recognized in *Lunataspis* are also revealed to be ancestral for the group at large, including the thoracetron being semicircular in shape and possessing a margin devoid of tergopleural spine projections. Two segments anterior to the thoracetron are freely articulating and the ancestral condition of the postabdomen is also shown to be multisegmented. The possession of a marginal rim to the thoracetron demarcated by a dorsal groove is, however, shown to be an autapomorphy of *Lunataspis* and not part of the xiphosuran ground pattern.

Determining the total number of body segments in *Ciurcalimulus* is difficult owing to the heavily exfoliated nature of the specimen, but a tentative count broadly conforms with the condition observed in *Lunataspis*, which possesses five segments in the postabdomen in addition to six fused segments within the thoracetron and the two freely articulating segments between the thoracetron and prosoma [1,10]. An opisthosoma comprising 13 segments may be ancestral for crown-group euchelicerates (including Xiphosura, Eurypterida and Arachnida [52]) and retained in the earliest horseshoe crabs before subsequent reduction in xiphosurids [1,52]. The ancestral condition of the prosomal appendages is likely to be chelate; while *Lunataspis* preserves no prosomal appendages, the possible appendage in *Ciurcopterus* has a chelate aspect (although its identification is again tentative), and chelate endopods are considered to be ancestral for chasmataspidids [53] while being ubiquitous among xiphosurids [54]. Offacoliids also possess chelate endopods [55,56] within their biramous limbs that appear essentially identical to the endopods of xiphosurids and are likely ancestral for euchelicerates, with the endopod being lost some time prior to the origin of crown group euchelicerates. This is further supported by the apparent occurrence of chelate limbs in the Fezouata xiphosuran [11].

Perhaps the most unusual feature of *Ciurcalimulus*, the bifurcated telson, is also shared with offacolids [33,55,56]. The bifurcation is not likely to be directly homologous, however, as the telson of *Lunataspis* and xiphosurids is not bifurcated, and nor are those of the weinberginid and bunodid synziphosurines that resolve phylogenetically between offacolids and crown euchelicerates. The bifurcated telson of *Ciurcalimulus* is, therefore, considered to be an autapomorphy of the genus and may represent a case of parallelism or possibly a deep-seated character reversal. Bifurcated telsons are also occasionally observed in modern horseshoe crabs as the result of developmental malfunctions in response to an injury or other damage [57]. In such cases the resulting bifurcation is generally asymmetrical and the telson truncated; the neatness of the bifurcation in *Ciurcalimulus* and the length of the telson would suggest that the structure is a genuine characteristic of the species, although ideally further specimens preserving the bifurcation would be required to determine this with absolute certainty.

The Silurian age of *Ciurcalimulus* shows that Xiphosura retaining the general morphology observed among Ordovician taxa persisted beyond the end Ordovician mass extinction, suggesting the event had a limited impact on horseshoe crab evolution. It seems likely that xiphosurids may not have originated until after the close of the Ordovician. It is possible that xiphosurids evolved sometime in the Silurian and persisted at low diversity alongside forms such as *Ciurcalimulus* prior to the major radiation of xiphosurids toward the end of the Devonian; alternatively, xiphosurids may only have originated closer to their first appearance in the fossil record. Either way, the diversification of xiphosurids appears to be linked to the expansion of marginal and nonmarine habitats in the Late Devonian driven by the influx of terrestrial nutrients [58,59].

### (c) Early horseshoe crab biogeography

Over the course of their evolutionary history, horseshoe crabs attained a global distribution [2]; however, the first xiphosurids are known from the palaeocontinents of Laurussia and Siberia, while the oldest described horseshoe crabs are found in Laurentia. The additional discovery of *Ciurcalimulus* suggests that Laurentia was an important site of early horseshoe crab evolution, although it must be recognized that there is a strong historical bias in palaeontological research toward localities in Europe and former European colonies [60,61]. As such, Laurentia is likely to be more heavily sampled than other palaeocontinents such as Gondwana, an important consideration given that the oldest currently known horseshoe crab is an undescribed species from Morocco. With such a limited early Palaeozoic fossil record it is not possible to infer where horseshoe crabs originated with any certainty, although two possibilities present themselves. One is that xiphosurans originated in Laurentia, with the rarity of horseshoe crabs in the Ordovician and Silurian either a genuine indication of low diversity or perhaps the result of their inhabiting environments such as deeper-water habitats that are usually less conducive to preservation. Alternatively, the Laurentian species may represent dispersal from a largely unsampled Gondwanan record, as has been suggested for Ordovician eurypterids [62]. More discoveries will be required to further elucidate the early evolution of the group, but the description of *Ciurcalimulus*, bridging an 80 million-year gap in the horseshoe crab fossil record, is an important step towards this goal.

## Data Availability

The phylogenetic matrix is available in the online Morphobank database under the project code p1253 (http://morphobank.org/permalink/?P1253). Supplementary material is available online [63].

## References

[1] Lamsdell JC, Ocon SB. 2025 Segmentation in early Xiphosura and the evolution of the thoracetron. J. Paleontol. (10.1017/jpa.2024.31)

[2] Lamsdell JC. 2020 The phylogeny and systematics of Xiphosura. PeerJ **8**, e10431. (10.7717/peerj.10431) 33335810 PMC7720731

[3] Eldredge N. 1976 Rates of evolution revisited. Paleobiology **2**, 174–179. (10.1017/S0094837300003456)

[4] Fisher DC. 1984 The Xiphosurida: archetypes of bradytely? In Living fossils (eds N Eldredge, SM Stanley), pp. 196–213. New York, NY: Springer-Verlag. (10.1007/978-1-4613-8271-3_23)

[5] Kin A, Błażejowski B. 2014 The horseshoe crab of the genus Limulus: living fossil or stabilomorph? PLoS One **9**, e108036. (10.1371/journal.pone.0108036) 25275563 PMC4183490

[6] Lamsdell JC. 2016 Horseshoe crab phylogeny and independent colonizations of fresh water: ecological invasion as a driver for morphological innovation. Palaeontology **59**, 181–194. (10.1111/pala.12220)

[7] Lamsdell JC. 2021 A new method for quantifying heterochrony in evolutionary lineages. Paleobiology **47**, 363–384. (10.1017/pab.2020.17)

[8] Lamsdell JC. 2021 The conquest of spaces: exploring drivers of morphological shifts through phylogenetic palaeoecology. Palaeogeogr. Palaeoclimatol. Palaeoecol. **583**, 110672. (10.1016/j.palaeo.2021.110672)

[9] Rudkin DM, Young GA, Nowlan GS. 2008 The oldest horseshoe crab: a new xiphosurid from Late Ordovician Konservat‐Lagerstätten deposits, Manitoba, Canada. Palaeontology **51**, 1–9. (10.1111/j.1475-4983.2007.00746.x)

[10] Lamsdell JC, Isotalo PA, Rudkin DM, Martin MJ. 2023 A new species of the Ordovician horseshoe crab Lunataspis. Geol. Mag. **160**, 167–171. (10.1017/s0016756822000875)

[11] Van Roy P, Orr PJ, Botting JP, Muir LA, Vinther J, Lefebvre B, Hariri K, Briggs DEG. 2010 Ordovician faunas of Burgess Shale type. Nature **465**, 215–218. (10.1038/nature09038) 20463737

[12] Bicknell RDC, Lustri L, Brougham T. 2019 Revision of "Bellinurus" carteri (Chelicerata: Xiphosura) from the Late Devonian of Pennsylvania, USA. C. R. Palevol **18**, 967–976. (10.1016/j.crpv.2019.08.002)

[13] Bicknell RDC, Smith PM. 2021 Patesia n. gen., a new Late Devonian stem xiphosurid genus. Palaeoworld **30**, 440–450. (10.1016/j.palwor.2020.09.001)

[14] Lamsdell JC, Selden PA. 2017 From success to persistence: identifying an evolutionary regime shift in the diverse Paleozoic aquatic arthropod group Eurypterida, driven by the Devonian biotic crisis. Evolution **71**, 95–110. (10.1111/evo.13106) 27783385

[15] Kluessendorf J. 1994 Predictability of Silurian Fossil‐Konservat‐Lagerstätten in North America. Lethaia **27**, 337–344. (10.1111/j.1502-3931.1994.tb01584.x)

[16] Pollock C, Rexroad C. 1973 Conodonts from the Salina Formation and the upper parts of the Wabash Formation (Silurian) in north-central Indiana. Geol. Palaeontol. **7**, 77–92. (10.1130/GES025.1)

[17] Droste JB, Shaver RH. 1982 The Salina Group (Middle and Upper Silurian) of Indiana. IN Geol. Surv. Spec. Rep. **24**, 1–41. (10.5967/geny-tq53)

[18] Claypole EW. 1890 Palaeontological notes from Indianapolis (A.A.A.S.) Pterichthys—Castoroides—Eurysoma g. n. Am. Geol. **6**, 255–260.

[19] Clarke JM, Ruedemann R. 1912 The Eurypterida of New York. Mem. NY St. Mus. Nat. Hist. **14**, 1–439.

[20] Kjellesvig-Waering EN. 1948 Two new eurypterids from the Silurian of Indiana. J. Paleontol. **22**, 465–472.

[21] Kjellesvig-Waering EN. 1958 Some previously unknown morphological structures of Carcinosoma newlini (Claypole). J. Paleontol. **32**, 295–303.

[22] Tetlie OE, Briggs DEG. 2009 The origin of pterygotid eurypterids (Chelicerata: Eurypterida). Palaeontology **52**, 1141–1148. (10.1111/j.1475-4983.2009.00907.x)

[23] Tetlie OE, Brandt DS, Briggs DEG. 2008 Ecdysis in sea scorpions (Chelicerata: Eurypterida). Palaeogeogr. Palaeoclimatol. Palaeoecol. **265**, 182–194. (10.1016/j.palaeo.2008.05.008)

[24] LoDuca ST, Swinehart AL, LeRoy MA, Tetreault DK, Steckenfinger S. 2021 Codium-like taxa from the Silurian of North America: morphology, taxonomy, paleoecology, and phylogenetic affinity. J. Paleontol. **95**, 207–235. (10.1017/jpa.2020.85)

[25] Curnings E, Shrock R. 1927 The Silurian coral reefs of northern Indiana and their associated strata. IN Acad. Sci. Proc. **36**, 71–85.

[26] Gupta NS, Tetlie OE, Briggs DEG, Pancost RD. 2007 The fossilization of eurypterids: a result of molecular transformation. Palaios **22**, 439–447. (10.2110/palo.2006.p06-057r)

[27] Kilibarda Z, Doffin J. 2007 Mudcracks, bird’s-eye, and anhydrite in intertidal/supratidal Late Silurian Kokomo Limestone, Indiana. Proc. IN Acad. Sci. **116**, 1–10.

[28] Kaye TG, Falk AR, Pittman M, Sereno PC, Martin LD, Burnham DA, Gong E, Xu X, Wang Y. 2015 Laser-stimulated fluorescence in paleontology. PLoS One **10**, e0125923. (10.1371/journal.pone.0125923) 26016843 PMC4446324

[29] Lamsdell JC. 2013 Revised systematics of Palaeozoic ‘horseshoe crabs’ and the myth of monophyletic Xiphosura. Zool. J. Linn. Soc. **167**, 1–27. (10.1111/j.1096-3642.2012.00874.x)

[30] Selden PA, Lamsdell JC, Qi L. 2015 An unusual euchelicerate linking horseshoe crabs and eurypterids, from the Lower Devonian (Lochkovian) of Yunnan, China. Zool. Scr. **44**, 645–652. (10.1111/zsc.12124)

[31] Lamsdell JC, Briggs DEG, Liu HP, Witzke BJ, McKay RM. 2015 A new Ordovician arthropod from the Winneshiek Lagerstätte of Iowa (USA) reveals the ground plan of eurypterids and chasmataspidids. Sci. Nat. **102**, 63. (10.1007/s00114-015-1312-5) 26391849

[32] Lamsdell JC, McKenzie SC. 2015 Tachypleus syriacus (Woodward)—a sexually dimorphic Cretaceous crown limulid reveals underestimated horseshoe crab divergence times. Org. Divers. Evol. **15**, 681–693. (10.1007/s13127-015-0229-3)

[33] Lustri L, Gueriau P, Daley AC. 2024 Lower Ordovician synziphosurine reveals early euchelicerate diversity and evolution. Nat. Commun. **15**, 3803. (10.1038/s41467-024-48013-w) 38714651 PMC11076625

[34] Lustri L, Antcliffe JB, Gueriau P, Daley AC. 2024 New specimens of Bunaia woodwardi Clarke, 1919 (Euchelicerata): a new member of Offacolidae providing insight supporting the Arachnomorpha. R. Soc. Open Sci. **11**, 240499. (10.1098/rsos.240499) 39479250 PMC11524597

[35] Aria C, Caron JB. 2017 Mandibulate convergence in an armoured Cambrian stem chelicerate. BMC Evol. Biol. **17**, 261. (10.1186/s12862-017-1088-7) 29262772 PMC5738823

[36] Aria C, Caron JB. 2019 A middle Cambrian arthropod with chelicerae and proto-book gills. Nature **573**, 586–589. (10.1038/s41586-019-1525-4) 31511691

[37] Tanaka G, Hou X, Ma X, Edgecombe GD, Strausfeld NJ. 2013 Chelicerate neural ground pattern in a Cambrian great appendage arthropod. Nature **502**, 364–367. (10.1038/nature12520) 24132294

[38] Liu Y, Ortega-Hernández J, Zhai D, Hou X. 2020 A reduced labrum in a Cambrian great-appendage euarthropod. Curr. Biol. **30**, 3057–3061.(10.1016/j.cub.2020.05.085) 32589912

[39] Legg DA, Sutton MD, Edgecombe GD. 2013 Arthropod fossil data increase congruence of morphological and molecular phylogenies. Nat. Commun. **4**, 2485. (10.1038/ncomms3485) 24077329

[40] Lan T, Zhao Y, Zhao F, He Y, Martinez P, Strausfeld NJ. 2021 Leanchoiliidae reveals the ancestral organization of the stem euarthropod brain. Curr. Biol. **31**, 4397–4404.(10.1016/j.cub.2021.07.048) 34416180

[41] O’Leary MA, Kaufman SG. 2012 Morphobank 3.0: web application for morphological phylogenetics and taxonomy. See http://www.morphobank.org.

[42] Goloboff PA, Farris JS, Nixon KC. 2008 TNT, a free program for phylogenetic analysis. Cladistics **24**, 1096-0031.2008.00217.x. (10.1111/j.1096-0031.2008.00217.x)

[43] Congreve CR, Lamsdell JC. 2016 Implied weighting and its utility in palaeontological datasets: a study using modelled phylogenetic matrices. Palaeontology **59**, 447–462. (10.1111/pala.12236)

[44] Felsenstein J. 1985 Confidence limits on phylogenies: an approach using the bootstrap. Evolution **39**, 783–791. (10.1111/j.1558-5646.1985.tb00420.x) 28561359

[45] Gravenhorst JLC. 1843 Vergleichende zoologie [Comparative Zoology]. Breslau, Poland: Grass, Barth und comp.

[46] Heymons R. 1901 Die Entwicklungsgeschichte der Scolopender [The evolutionary history of the invertebrates]. Zoologica **13**, 1–244.

[47] Latreille P. 1802 Histoire naturelle, générale et particulière des crustacés et insectes. familles naturelles des genres [natural history, general and particular, of crustaceans and insects. natural families of genera]. vol. 3. Paris, France: F. Dufart. (10.5962/bhl.title.15764)

[48] Falk AR, Kaye TG, Zhou Z, Burnham DA. 2016 Laser fluorescence illuminates the soft tissue and life habits of the Early Cretaceous bird Confuciusornis. PLoS One **11**, e0167284. (10.1371/journal.pone.0167284) 27973609 PMC5156344

[49] McCoy VE, Brandt DS. 2009 Scorpion taphonomy: criteria for distinguishing fossil scorpion molts and carcasses. J. Arachnol. **37**, 312–320. (10.1636/sh09-07.1)

[50] Lamsdell JC. 2011 The eurypterid Stoermeropterus conicus from the lower Silurian of the Pentland Hills, Scotland. Palaeontogr. Soc. Monogr. **165**, 1–84. (10.1080/25761900.2022.12131816)

[51] Anderson LI, Selden PA. 1997 Opisthosomal fusion and phylogeny of Palaeozoic Xiphosura. Lethaia **30**, 19–31. (10.1111/j.1502-3931.1997.tb00440.x)

[52] Dunlop JA, Lamsdell JC. 2017 Segmentation and tagmosis in Chelicerata. Arthropod Struct. Dev. **46**, 395–418. (10.1016/j.asd.2016.05.002) 27240897

[53] Lamsdell JC, Gunderson GO, Meyer RC. 2019 A common arthropod from the Late Ordovician Big Hill Lagerstätte (Michigan) reveals an unexpected ecological diversity within Chasmataspidida. BMC Evol. Biol. **19**, 8. (10.1186/s12862-018-1329-4) 30621579 PMC6325806

[54] Bicknell RDC, Birch SA, Charbonnier S, Sautereau F, Hitij T, Campione NE. 2019 On the appendicular anatomy of the xiphosurid Tachypleus syriacus and the evolution of fossil horseshoe crab appendages. Sci. Nat. **106**, 38. (10.1007/s00114-019-1629-6) 31209559

[55] Sutton MD, Briggs DEG, Siveter DJ, Siveter DJ, Orr PJ. 2002 The arthropod Offacolus kingi (Chelicerata) from the Silurian of Herefordshire, England: computer based morphological reconstructions and phylogenetic affinities. Proc. R. Soc. Lond. B **269**, 1195–1203. (10.1098/rspb.2002.1986) PMC1691018 12065034

[56] Briggs DEG, Siveter DJ, Siveter DJ, Sutton MD, Garwood RJ, Legg D. 2012 Silurian horseshoe crab illuminates the evolution of arthropod limbs. Proc. Natl Acad. Sci. USA **109**, 15702–15705. (10.1073/pnas.1205875109) 22967511 PMC3465403

[57] Bicknell RDC, Pates S, Botton ML. 2018 Abnormal xiphosurids, with possible application to Cambrian trilobites. Palaeontol. Electron. **2018**, 21.2.19A. (10.26879/866)

[58] Buatois LA, Mangano GM, Genise JF, Taylor TN. 1998 The ichnologic record of the continental invertebrate invasion: evolutionary trends in environmental expansion, ecospace utilization, and behavioral complexity. Palaios **13**, 217–240. (10.2307/3515447)

[59] Hughes ES, Lamsdell JC. 2021 Discerning the diets of sweep-feeding eurypterids: assessing the importance of prey size to survivorship across the Late Devonian mass extinction in a phylogenetic context. Paleobiology **47**, 271–283. (10.1017/pab.2020.18)

[60] Monarrez PM *et al*. 2022 Our past creates our present: a brief overview of racism and colonialism in Western paleontology. Paleobiology **48**, 173–185. (10.1017/pab.2021.28)

[61] Raja NB, Dunne EM, Matiwane A, Khan TM, Nätscher PA, Ghilardi AM, Chattopadhyay D. 2022 Colonial history and global economics distort our understanding of deep-time biodiversity. Nat. Ecol. Evol. **6**, 145–154. (10.1038/s41559-021-01608-8) 34969991

[62] Lamsdell JC, Hoşgör İ, Selden PA. 2013 A new Ordovician eurypterid (Arthropoda: Chelicerata) from southeast Turkey: evidence for a cryptic Ordovician record of Eurypterida. Gondwana Res. **23**, 354–366. (10.1016/j.gr.2012.04.006)

[63] Lamsdell JC. 2025 Supplementary material from: The first Silurian horseshoe crab reveals details of the xiphosuran ground plan. Figshare. (10.6084/m9.figshare.c.7836714)40527460

